# The Construction and Validation of a Machine Learning Model for Major Depressive Disorder Based on Astrocytes and Mitochondrial Dynamics

**DOI:** 10.3390/cimb48101075

**Published:** 2026-10-17

**Authors:** Shi Lin, Gaoyuan Wang, Xiaoqin Shao, Yinghui Mo, Yating Song, Zuyi Lu, Jiangli Zhou, Zhiqing Lu, Quan Wen, Yuanning Zeng, Qian Wang

**Affiliations:** 1 Research Centre of Basic Integrative Medicine, School of Basic Medical Science, Guangzhou University of Chinese Medicine, Guangzhou 510006, China; 2 Guangdong Engineering Technology Research Center for Standardized Processing of Chinese Materia Medica, School of Chinese Materia Medica, Guangdong Pharmaceutical University, Guangzhou 510006, China

**Keywords:** major depressive disorder, astrocytes, mitochondrial dynamics, machine learning, hexokinase 2, ribosomal protein S7

## Abstract

Background: Astrocytes play an important role in depression. However, their specific mechanisms, especially their subcellular mechanisms, remain unclear. Objective: We aimed to identify key genes associated with mitochondrial dynamics and astrocytes in Major Depressive Disorder (MDD), construct a diagnostic prediction model, and elucidate their regulatory mechanisms in neuroinflammation. Methods: Depression-related transcriptome datasets were retrieved from the GEO database. Overlapping differentially expressed genes between mitochondrial dynamics-related and astrocytes-associated genes were screened, followed by functional enrichment analysis. Machine learning algorithms were employed to establish a diagnostic model and identify hub genes. An LPS-induced mouse depression model was established, and depressive-like phenotypes were assessed through behavioral tests. Hub gene expression was validated by using quantitative real-time PCR and Western blotting. Results: A machine learning model with favorable diagnostic performance was constructed. LPS markedly upregulated hexokinase 2 (HK2) and ribosomal protein S7 (RPS7) expression in the prefrontal cortex and hippocampus, suggesting that glycolytic metabolism and ribosomal function contribute to LPS-induced neuropathological changes. Conclusions: HK2 and RPS7 were identified and validated as crucial hub genes responsive to neuroinflammation. The combined action of mitochondrial dynamics and astrocyte metabolic abnormalities may play a key role in depression’s pathogenesis, providing novel candidate biomarkers and theoretical support for the molecular diagnosis of MDD and anti-inflammatory targeted therapy.

## 1. Introduction

Major Depressive Disorder (MDD) is a severe mental disorder with high incidence and recurrence rates [1]; it is characterized by a persistent low mood lasting more than 2 weeks, recurrent thoughts of death and suicide, feelings of worthlessness, social isolation, and anhedonia [2,3,4]. The etiology of depression is complex, involving genetic and psychosocial factors such as stress, illness, post-traumatic stress, social isolation, social trauma, and abuse, among others [5,6,7,8]. According to the World Health Organization (WHO, 2026), approximately 322 million people suffer from depression worldwide, accounting for 4% of the global population, with an adult prevalence of 5.2%; in 2021, approximately 727,000 people died by suicide worldwide, with depression being an important contributing factor [9,10].

The pathogenesis of MDD is complex, involving genetic variations, abnormalities in neural plasticity, dysregulated inflammatory responses, and post-transcriptional regulatory disorders [1]. Neuroinflammation is a core pathological mechanism in the onset and progression of various central nervous system (CNS) disorders, characterized by abnormal activation of glial cells and excessive release of pro-inflammatory factors; these subsequently lead to neuronal damage and apoptosis, disrupting the homeostasis of the brain microenvironment [3,11]. Numerous studies have recently confirmed the association between neuroinflammation and depression. In clinical research, levels of pro-inflammatory cytokines such as C-reactive protein (CRP), interleukin-1β (IL-1β), interleukin-6 (IL-6), and tumor necrosis factor-α (TNF-α) were significantly elevated in the peripheral and central nervous system of patients with MDD; however, antidepressant treatment reduced the expression of pro-inflammatory cytokines in the CNS [4,12,13,14,15,16]. Collectively, these findings suggest neuroinflammation as a key pathological factor in the development of *MDD*.

Astrocytes, the most abundant glial cells in the *CNS*, help maintain neuronal metabolic homeostasis, provide nutritional support, and play a key role in regulating neuroinflammation [17]. Their activation facilitates the release of pro-inflammatory factors [11,18], mediating inflammation-induced neurotoxicity, and their dysfunction closely relates to the pathological progression of various neurodegenerative diseases and mental disorders [11,19,20,21,22]. In previous studies, various anti-inflammatory agents could ameliorate depressive symptoms by suppressing neuroinflammation. For instance, celecoxib, a selective COX-2 inhibitor, attenuates prostaglandin synthesis through cyclooxygenase inhibition, thereby reducing the release of pro-inflammatory cytokines (IL-1β and TNF-α) and reversing stress-induced depressive behaviors. Additionally, simvastatin mitigates inflammatory mediators by inhibiting HMG-CoA reductase, suppresses microglial activation and the NF-κB signaling pathway, and has been shown to restore hippocampal BDNF levels and improve depressive behaviors in animal models [23]. Lipopolysaccharide (LPS), a major component of the cell wall of Gram-negative bacteria [24,25], actives glial cells and triggers neuroinflammatory responses activating the Toll-like receptor 4 (TLR4) signaling pathway [11,16,26]. It is a classic inducer in constructing models of inflammation and depression.

With the rapid advancement of bioinformatics and high-throughput sequencing technologies, mining disease-critical molecules from public databases has become essential in investigating the mechanisms of psychiatric disorders [27,28]. The Gene Expression Omnibus (GEO) database provides abundant data resources for performing transcriptomic analyses of depression. Differentially expressed gene screening, functional enrichment analysis, and machine learning [29] allow core genes closely associated with the disease to be efficiently identified, offering novel insights to enable early diagnosis and targeted research on depression. Mitochondrial dynamics is as a critical process for maintaining cellular structure and energy metabolism; when disrupted, it can lead to insufficient neuronal energy supply, enhanced oxidative stress, and the activation of apoptotic pathways, thereby contributing to neuroinflammation and mood regulation disorders [30,31,32]. Astrocytes participate in neurotransmitter metabolism, regulate synaptic plasticity, and modulate the inflammatory response; their functional impairment is closely associated with depression, and astrocyte-specific molecular alterations in the prefrontal cortex have been shown to causally contribute to stress-induced behavioral deficits in animal models [33,34,35,36]. However, studies combining mitochondrial dynamics-related genes with astrocyte-associated genes for screening key molecules in depression remain scarce, and the interactive regulatory mechanisms between these two factors in depression remain unclear.

Based on the aforementioned research status, we integrated depression datasets from the GEO database to screen differentially expressed genes associated with mitochondrial dynamics and astrocytes (MD-AST DEGs). Multiple machine learning algorithms were employed to construct a diagnostic prediction model for depression; this was subsequently validated through behavioral and molecular assessments in a Lipopolysaccharide-induced mouse model of depression and anxiety. The objective of this study was to identify stable and reliable molecular biomarkers for depression; elucidate the synergistic role of mitochondrial dynamics, astrocytes, and metabolic abnormalities in the pathogenesis of depression; and provide experimental evidence and theoretical foundations for mechanistic investigations, early diagnosis, and targeted therapeutic strategies for depression.

## 2. Materials and Methods

### 2.1. Data Preprocessing

We searched gene expression microarray datasets, which featured patients with Major Depressive Disorder (MDD) and healthy controls (HCs), on the Gene Expression Omnibus (GEO, https://www.ncbi.nlm.nih.gov/geo/), using the keyword “major depressive disorder” and the species filter “*Homo sapiens*”. In this study, five datasets were selected: GSE98793 (platform GPL570), which includes data from 64 MDD patients without comorbid anxiety and 64 HCs; GSE32280 (platform GPL57), including 8 medication-free MDD patients and 8 HCs; GSE19738 (platform GPL6848), including 33 MDD patients and 34 HCs; GSE76826 (platform GPL17077), including 20 MDD patients and 12 HCs; and GSE53987 (platform GPL570), including 17 MDD patients and 19 HCs Table S1. We selected the GSE98793, GSE32280, and GSE19738 datasets as the training sets and used the “combat” function from the “sva” package in RStudio (version 2025.05.0 Build 496) to perform batch effect removal on them. This allowed us to reduce errors caused by differences in reagents, procedures, and sequencing platforms. Grouping was conducted using the “data. table” package, and data reading, preprocessing, differential analysis, and visualization were performed using the “tinyarray” package. GSE76826 and GSE53987 were used as external validation datasets for subsequent analyses.

GeneCards (https://www.genecards.org/, accessed in June 2026) is a comprehensive database of information on all annotated and predicted human genes [37]. We separately searched for “fusion”, “fission”, “biogenesis”, “mitophagy” and “astrocyte” in GeneCards, filtering for “Protein-Coding” genes and correlation scores > 1. In total, we obtained 8625 genes related to mitochondrion dynamics (MDGs) and 6857 astrocyte-associated genes (AAGs). Duplicate genes were found in the initial search results of the MDGs; after deduplication, 6481 unique MDGs were obtained.

### 2.2. Identification of Differentially Expressed Genes Related to Mitochondrial Dynamics and Astrocytes

We performed differential expression analysis of genes between MDD patients and HC in the training set using the “limma” package in RStudio version 2025.05.0; thresholds for differential gene selection were set to *p* < 0.05 and |LogFC (fold change)| > 0.2 [38,39]. A volcano plot was created using the “ggplot2” package. To identify differentially expressed genes related to mitochondrial dynamics and astrocytes (MD-AST DEGs), we performed an intersection analysis of MDGs, AAGs, and DEGs using the ‘Venn’ module in MicroBioinformatics (https://www.bioinformatics.com.cn/).

### 2.3. Characteristic Gene Expression and Functional Enrichment Analysis

We generated boxplots displaying the expression of 18 MD-AST DEGs in the training set using the “ggpubr” package, and their correlation heatmaps were plotted using the “corrplot” package. Using the ‘clusterProfiler’ package, we performed functional enrichment analyses of these genes based on Gene Ontology (GO) and the Kyoto Encyclopedia of Genes and Genomes (KEGG); the top 6 pathways were selected based on the ascending order of p.adjust. The results of these analyses were visualized using the “GOplot” and “enrichplot” packages.

### 2.4. Machine Learning Algorithms for Screening Key Central Genes

Eight machine learning algorithms were employed to further screen for hub MD-AST DEGs: Random Forest (RF), a Support Vector Machine (SVM), a Generalized Linear Model (GLM), a Gradient Boosting Machine (GBM), k-Nearest Neighbors (KNN), a (Feed-forward) Neural Network (NNET), the Least Absolute Shrinkage and Selection Operator (LASSO), and a Decision Tree (DT). All model training, algorithm comparison, and feature selection were confined to the merged peripheral-blood training set, and cross-validation was deliberately not employed: instead, three completely independent external cohorts (GSE76826 and GSE53987 (PFC and hippocampus), which were never involved in training or feature selection) served as the primary safeguard against overfitting, constituting a more stringent test of generalizability than cross-validation; however, we acknowledge that in-sample training-set evaluation carries an optimistic bias. All algorithms were run with the default hyperparameters of the corresponding R packages, without targeted tuning, as our goal was to identify cross-algorithm-stable biomarker candidates rather than to maximize single-classifier performance in small-sample data. Training-set performance was compared via ROC/AUC (“pROC”), residual diagnostics (“DALEX” and “ingredients”), and permutation-based feature importance. Based on these evaluations, GLM, LASSO, RF-RFE, and SVM-RFE were selected; the latter three perform embedded feature selection (L1-regularized shrinkage for LASSO; Recursive Feature Elimination for RF-RFE and SVM-RFE), and the intersection of the four algorithms yielded seven hub MD-AST DEGs (AKR1C3, CBL, FKBP5, HK2, RORA, RPS7, and SIAH2) Tables S2 and S3.

### 2.5. Construction and Validation of a Diagnostic Prediction Model Based on Characteristic Genes

In order to analyze the effectiveness of the hub MD-AST DEGs in diagnosing MDD, logistic regression analysis was conducted using the “RMS” package, and we established a diagnostic prediction model. A nomogram was constructed based on these DEGs, and calibration curves were drawn to evaluate the diagnostic performance. For the same purpose, the relevant ROC curve was plotted using the “pROC’” package for the training set. Additional ROC curves were plotted using the external validation sets, GSE76826 and GSE53987, to further verify the predictive performance of the model based on these DEGs. Additionally, boxplots were generated using the “ggplot2” package to validate the DEGs’ expression levels.

### 2.6. Identification of Central Feature Gene-Related Transcription Factors and MicroRNAs

NetworkAnalyst (https://www.networkanalyst.ca/) is a comprehensive online analysis platform with well-established statistical workflows and advanced data visualization capabilities [40]. Transcription factors (TFs) are a diverse family of proteins that can bind to gene-specific sequences, regulating the activation or repression of gene transcription, which is crucial for normal organismal development and disease responses. MicroRNAs (miRNAs) can regulate the translation of target genes into proteins, thereby controlling gene expression. We used the JASPAR database to analyze the interactions between transcription factors and the hub MD-AST DEGs and used the TarBase database (through the online NetworkAnalyst platform) for those between miRNAs and the DEGs. Finally, the predicted interactions between TFs and genes, as well as between genes and miRNAs, were visualized using Cytoscape (version 3.10.3).

### 2.7. Construction of Animal Models for Depression

All animal procedures were strictly conducted in accordance with the Guide for the Care and Use of Laboratory Animals. Male C57BL/6J mice (7 weeks of age) were purchased from the Guangdong Provincial Medical Laboratory Animal Center (SCXK (YUE 2022-0002, Guangzhou, China) and housed in the Specific Pathogen-Free (SPF) Experimental Animal Center of the Guangzhou University of Chinese Medicine. The mice were maintained under controlled environmental conditions with a relative temperature of 24 °C, relative humidity of 50–60%, and a 12-h light/dark cycle. After a 3-day acclimatization period with ad libitum access to food and water, the mice were randomly divided into a control group and a Lipopolysaccharide (LPS) (Aladdin, L118716, Shanghai, China) model group. Mice in the control group received intraperitoneal injections of normal saline for 7 consecutive days, whereas those in the model group were administered 0.5 mg/kg of LPS for 7 consecutive days. All procedures were approved by the Laboratory Animal Ethics Committee of the Guangzhou University of Chinese Medicine (Approval Number: 20251027016). Behavioral experiments were conducted 24 h after the final LPS injection. Prior to each behavioral test, the mice were allowed to acclimate for at least 1 h. Apparatus was cleaned with 75% ethanol after each session. All recording and analysis software was provided by Shanghai Jiliang Software Technology Co., Ltd. (Shanghai, China).

### 2.8. Behavioral Tests

#### 2.8.1. Open Field Test

The open field test (OFT) was used to assess locomotor activity and anxiety-like behavior, as previously described. The mice were placed in the center of an open field (40 × 40 × 40 cm) and allowed to freely explore for 30 min. The aforementioned recording and analysis software (Shanghai Jiliang Software Technology Co, Ltd.) was used to record the total distance traveled by the mice over 30 min and the time spent in the central and peripheral zones within the first 5 min.

#### 2.8.2. Rotarod

A rotarod (RR) was used to assess locomotor activity according to previous descriptions. After 30 s of adaptation on the rotarod (diameter 3 cm), the rotation speed was gradually increased from 4 to 40 r/min, and the time taken to fall from the rotarod was recorded for each mouse (Shanghai Jiliang Software Technology Co, Ltd.).

#### 2.8.3. Elevated Plus Maze

An elevated plus maze (EPM) was utilized to assess anxiety-like behavior, as previously described. The apparatus consists of two opposing open arms (30 × 5 cm), two opposing closed arms (30 × 5 × 15 cm), and a central platform (5 × 5 cm), placed 30 cm from the ground. The number of entries and the duration of stay in the open and closed arms within 5 min were recorded and analyzed using a video recording system(Shanghai Jiliang Software Technology Co, Ltd.).

#### 2.8.4. Tail Suspension Test

As previously described, the tail suspension test (TST) was employed to evaluate depressive behavior. Each mouse’s tail was suspended 35 cm above the ground. The experiment lasted a total of 6 min, and the time spent immobile by each mouse during the last 4 min was recorded. Activity was automatically monitored and recorded using the relevant equipment. We recorded and analyzed idle time using the DigBehv Animal Behavior Analysis System (version 2.1.0).

#### 2.8.5. Forced Swim Test

The forced swim test (FST) was conducted in a glass cylinder filled with 30 cm of water (25 ± 1 °C) to evaluate depressive-like behavior, as previously described. Mice were placed in the cylinder for 6 min, and time spent immobile within the last 4 min was recorded using the DigBehv Animal Behavior Analysis System (version 2.1.0).

### 2.9. Real-Time Quantitative Pcr

RNA was extracted from the prefrontal cortex (PFC) and hippocampus (HIP) of LPS-treated mice using Trizol (Takara, 9108, Shiga, Japan). cDNA was then synthesized using a reverse transcription kit (Takara, RR037A, Shiga, Japan) and amplified using SYBR (Takara, RR820A, Shiga, Japan) for RT-qPCR. The primer sequences used in this study are listed in Table 1.

### 2.10. Western Blot

Tissue samples from the PFC and HIP were homogenized on ice using RIPA lysis buffer supplemented with 1% phenylmethylsulfonyl fluoride (PMSF) for protein extraction. Stacking and separating gels were prepared, and denatured protein samples were loaded. Electrophoresis was initially performed at 80 V; the voltage was subsequently adjusted to 110 V once the proteins had entered the separating gel. Following electrophoresis, proteins were transferred onto a membrane at 250 mA for 90 min in an ice bath. The membrane was then blocked with 5% skim milk at room temperature for 1.5 h, followed by three washes with TBST for 5 min each. Primary antibodies were incubated at 4 °C for 12–16 h. After three washes with TBST, the membrane was incubated with secondary antibodies at room temperature for 1 h, followed by three additional washes with TBST. Protein bands were visualized using enhanced chemiluminescence, and band intensities were quantified using ImageJ software(version 2.14.0). The gray values of the target protein bands were normalized to those of GAPDH.

### 2.11. Statistical Analysis

All experimental data were analyzed using GraphPad Prism 10.3.1 and R 4.5.0 software, and the results are expressed as the mean ± the standard error of the mean (mean ± SEM). Comparisons between two groups were performed using non-parametric tests, while comparisons among multiple groups were conducted using one-way analysis of variance (one-way ANOVA). *p*-values less than 0.05 were considered statistically significant.

## 3. Results

### 3.1. Identification of Md-Ast Degs Between Mdd Patients and Healthy Controls

**Figure 1:**
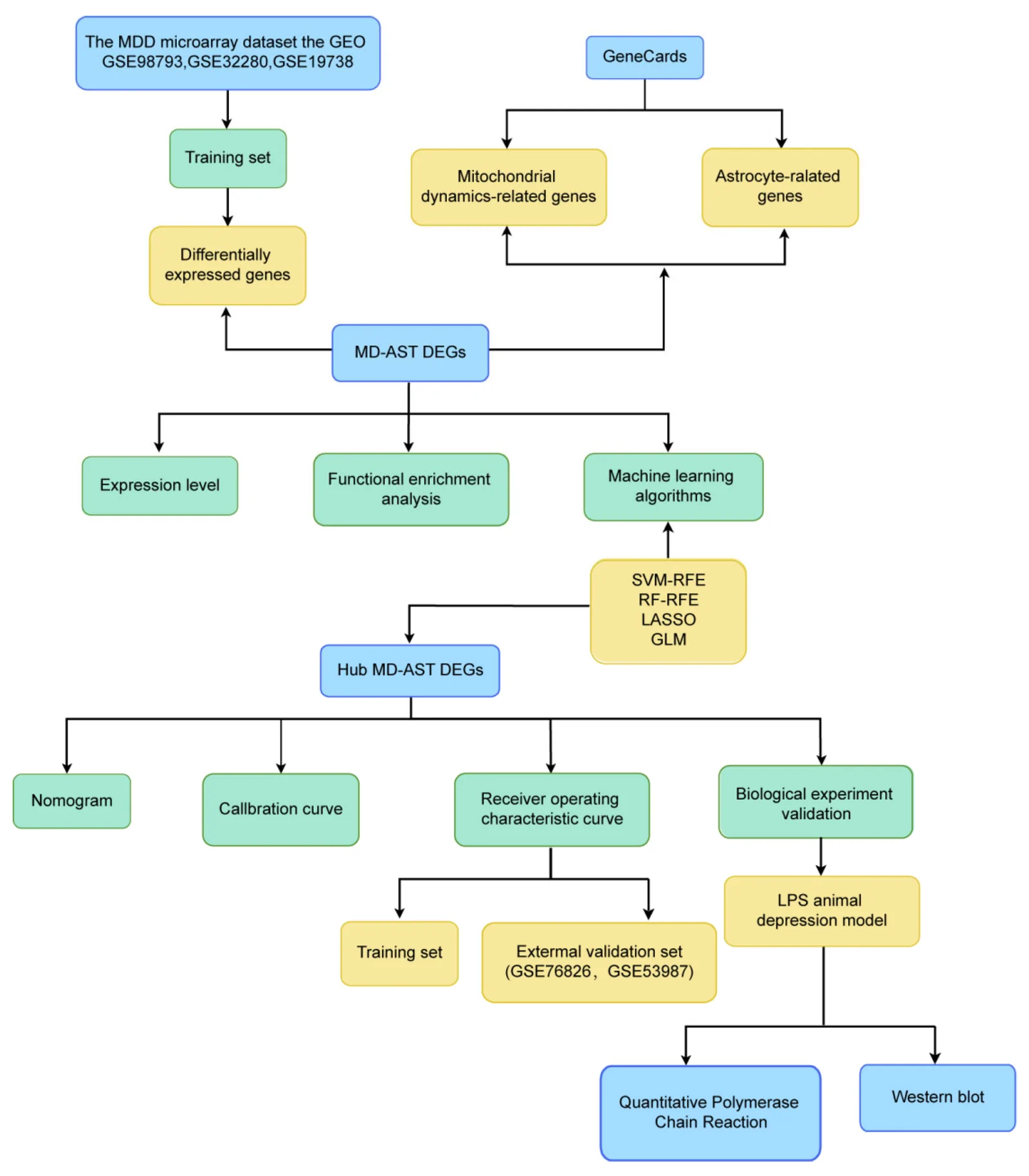
Research flow chart. MD-AST DEGs, Mitochondrial Dynamics and Astrocyte-Related Differentially Expressed Genes. GEO, Gene Expression Omnibus Database. MDD, Major Depressive Disorder. SVM-RFE, Support Vector Machine-Recursive Feature Elimination. RF-RFE, Random Forest-Recursive Feature Elimination. LASSO, Least Absolute Shrinkage and Selection Operator. GLM, Generalized Linear Model. LPS, Lipopolysaccharide. Arrows indicate the direction of the analysis workflow.

**Figure 2:**
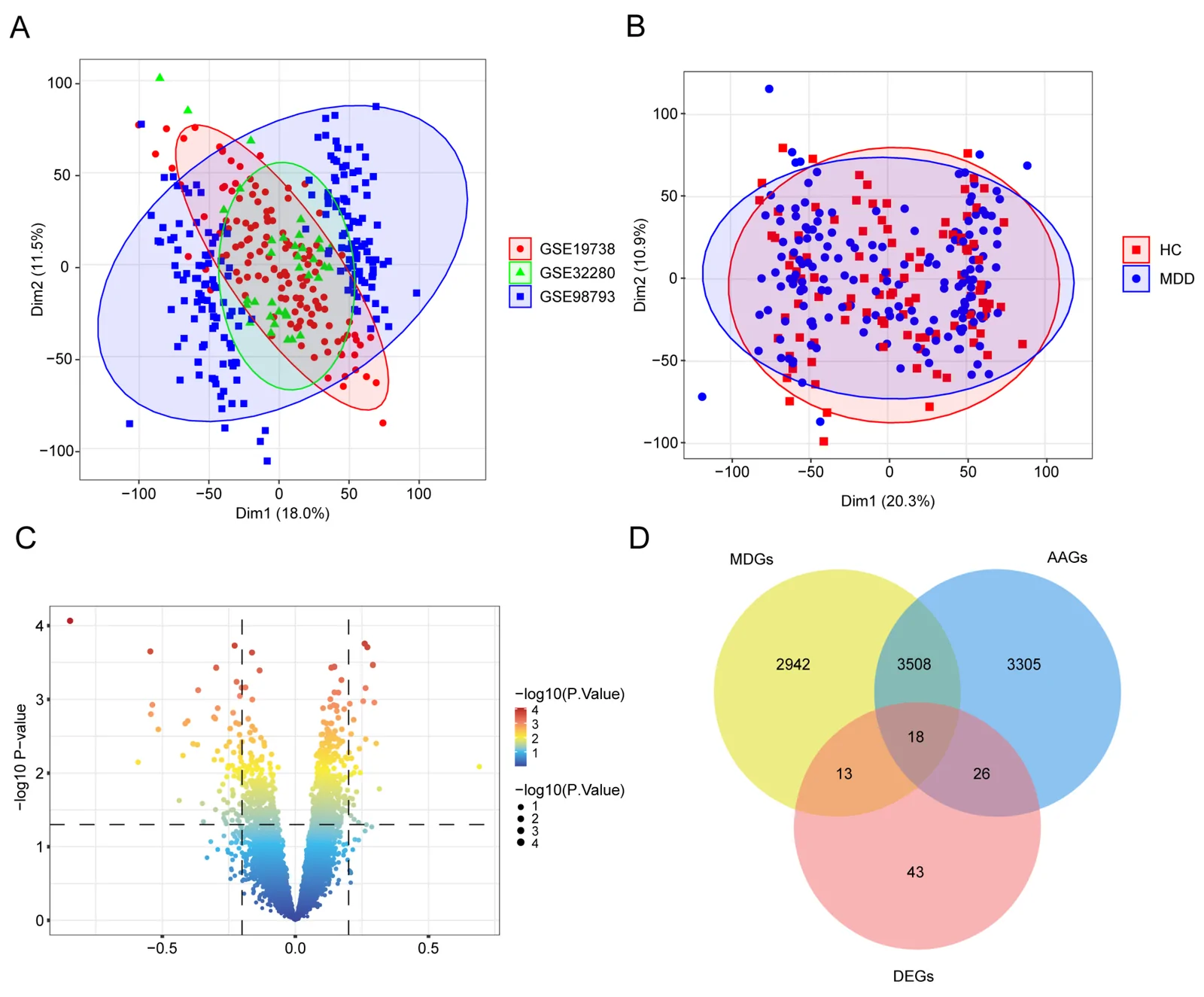
The identification of MD-AST DEGs. (**A**) PCA plots of all samples in the three datasets after removing batch effects. (**B**) PCA plots of HC and MDD samples from the three datasets after removing batch effects. (**C**) The volcano plots of DEGs in the training set. (**D**) The Venn diagram of the intersection of MDGs, AAGs, and DEGs. MDGs, Mitochondrial Dynamics-Related Genes. AAGs, Astrocyte-Associated Genes. DEGs, Differentially Expressed Genes. HC, Healthy Control. MDD, Major Depressive Disorder.

A flow chart of this research is shown in Figure 1. GSE98793, GSE32280, and GSE19738, featuring samples from patients with depression and healthy controls, were merged after removing batch effects to obtain the processed training set (Figure 2A,B and Supplementary Figure S1). A total of 100 DEGs were identified through differential expression analysis; 27 were upregulated and 73 downregulated (Figure 2C). Furthermore, 6553 MDGs and 6857 AAGs intersected with the 100 DEGs, resulting in 18 MD-AST DEGs (Figure 2D).

### 3.2. The Expression and Functional Enrichment Analysis of Md-Ast Degs

**Figure 3:**
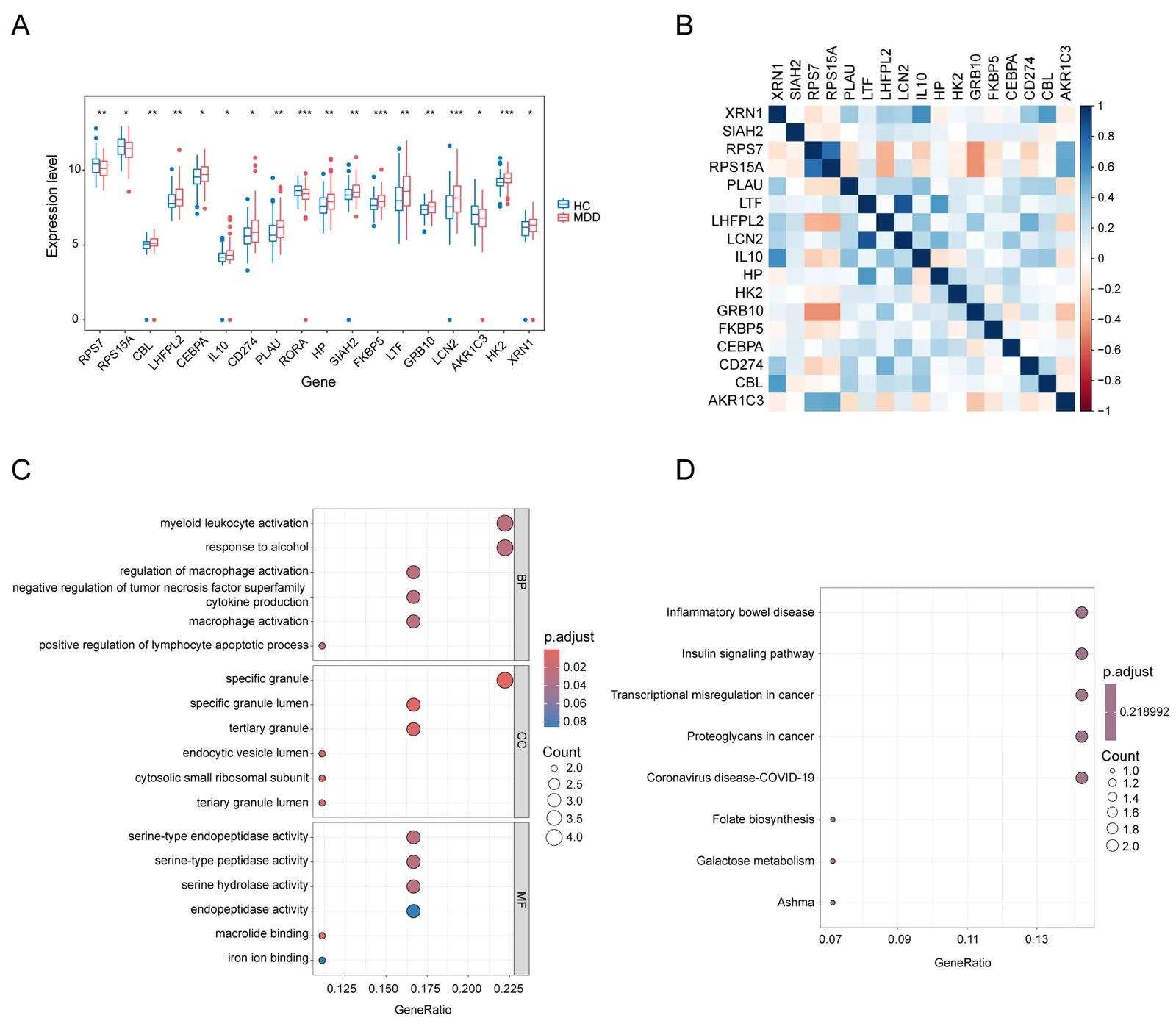
The expression and functional analysis of MD-AST DEGs. (**A**) The expression of MD-AST DEGs in the training set. (**B**) The heatmap of the correlation between MD-AST and DEGs. (**C**) Enriched bubble plots for KEGG functional analysis of MD-AST and DEGs. (**D**) Enriched bubble plots for GO functional analysis of MD-AST DEGs. * *p* < 0.05, ** *p* < 0.01, and *** *p* < 0.001. RPS7, Ribosomal Protein S7. RPS15A, Ribosomal Protein S15a. CBL, Casitas B-lineage Lymphoma Proto-Oncogene. LHFPL2, LHFPL Tetraspan Subfamily Member 2. CEBPA, CCAAT Enhancer Binding Protein Alpha. IL10, Interleukin 10. CD274, CD274 Molecule. PLAU, Plasminogen Activator, Urokinase. RORA, RAR Related Orphan Receptor A. HP, Haptoglobin. SIAH2, Siah E3 Ubiquitin Protein Ligase 2. FKBP5, FKBP Prolyl Isomerase 5. LTF, Lactotransferrin. GRB10, Growth Factor Receptor Bound Protein 10. LCN2, Lipocalin 2. AKR1C3, Aldo-Keto Reductase Family 1 Member C3. HK2, Hexokinase 2. XRN1, Chromatin-Binding Exonuclease XRN1.

In the MDD group, the expression of 18 MD-AST DEGs was upregulated and that of 4 MD-AST DEGs was downregulated, all of which were statistically significant (Figure 3A). Among the 18 MD-AST DEGs, RPS15A was most strongly negatively correlated with GRB10, RPS7 was most strongly negatively correlated with GRB10, and RPS15A was most strongly positively correlated with RPS7 (Figure 3B). KEGG and GO functional enrichment analyses were subsequently performed for these genes. Enriched biological processes in the GO enrichment analysis included myeloid leukocyte activation, negative regulation of cytokine production in the tumor necrosis factor superfamily, and macrophage activation (Figure 3C). Cellular components included specific granules, the specific granule lumen, and tertiary granules. Molecular functions included serine-type endopeptidase activity, serine-type peptidase activity, and serine hydrolase activity. In the KEGG enrichment analysis, the genes were primarily enriched in the insulin signaling pathway and galactose metabolism, among others (Figure 3D).

### 3.3. Eight Machine Learning Algorithms Identify Hub Md-Ast Degs

**Figure 4:**
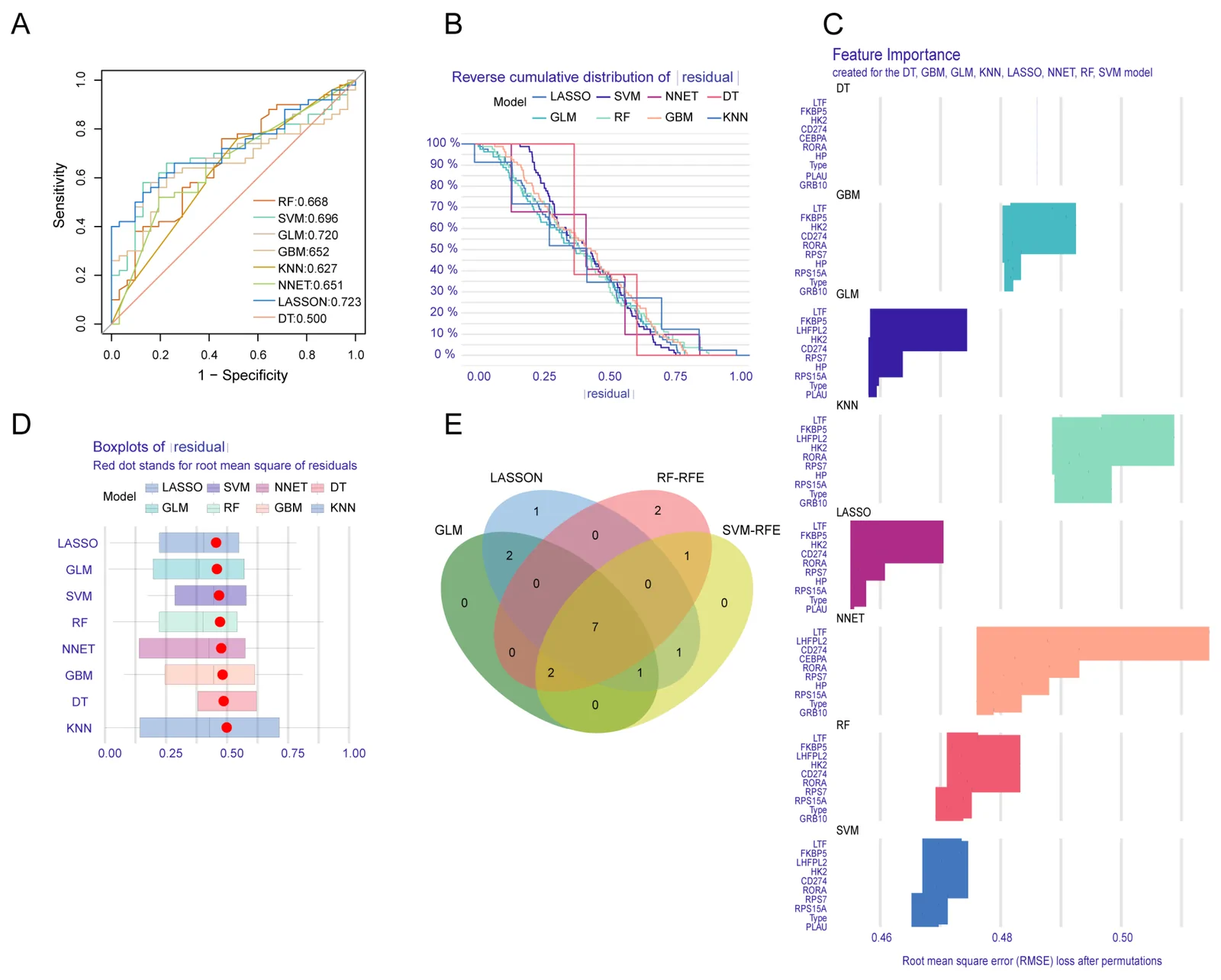
The performance evaluation of eight machine learning algorithms. (**A**) ROC curves of eight machine learning algorithms. (**B**) Inverse cumulative distribution function of absolute residuals. (**C**) Boxplots of absolute residuals. (**D**) The performance evaluation of eight machine learning algorithms. (**E**) Venn diagram of four machine learning algorithms used to screen hub MD-AST DEGs. DT, Decision Tree. GBM, Gradient Boosting Machine. GLM, Generalized Linear Model KNN, k-Nearest Neighbors. LASSO, Least Absolute Shrinkage and Selection Operator. NNET, (Feed-Forward) Neural Network. RF, Random Forest. SVM, Support Vector Machine.

We applied eight machine learning algorithms and compared the classification capabilities of different models using receiver operating characteristic (ROC) curves. Among them, LASSO exhibited the highest AUC value, the most stable predictive accuracy, and the strongest discriminative power; the GLM also performed well, with discriminative ability close to that of LASSO. The SVM and RF showed moderate performance, while the NNET and KNN performed moderately. The DT demonstrated performance comparable to random guessing, indicating limited utility in this context (Figure 4A). We compared the prediction errors of different models using residual reverse cumulative distribution plots. The LASSO curve was the farthest to the left and maintained a high cumulative proportion even with small residuals, indicating only minor errors in most of its predictions; hence, its predictive accuracy was the most stable. The GLM performed similarly to LASSO. The SVM, NNET, RF, GBM, and KNN had moderate error distributions and overall prediction accuracy, while the DT showed the largest prediction errors and the worst performance (Figure 4B). Each model’s feature importance was evaluated using permutation-based mean squared error loss. The NNET had the highest scores in this domain, indicating the strongest dependency on features, followed by KNN. The GBM, GLM, LASSO, RF, and SVM scored moderately here, with concentrated and stable feature contributions. The DT scored lowest, with almost no apparent color blocks in the visualization, indicating the weakest dependency on features (Figure 4C). The distribution of prediction errors was compared using residual boxplots. The red dots for the GLM and LASSO were furthest to the left, and the boxes were relatively narrow, indicating small overall errors and stable predictive performance. RF, the SVM, and the DT had even narrower box widths, with the same implications. The NNET and GBM had moderately wide boxes, indicating stable error performance, while KNN’s red dots were furthest to the right and had the widest boxes, indicating the largest overall errors and the poorest predictive stability (Figure 4D). A comprehensive analysis showed that the GLM, LASSO, RF-RFE, and SVM-RFE performed the best. Thus, we took the intersecting results from these four algorithms, yielding seven hub MD-AST DEGs: AKR1C3, CBL, FKBP5, HK2, RORA, RPS7, and SIAH2 (Figure 4E).

### 3.4. Construction and Validation of a Diagnostic Model Based on Hub Md-Ast Degs

**Figure 5:**
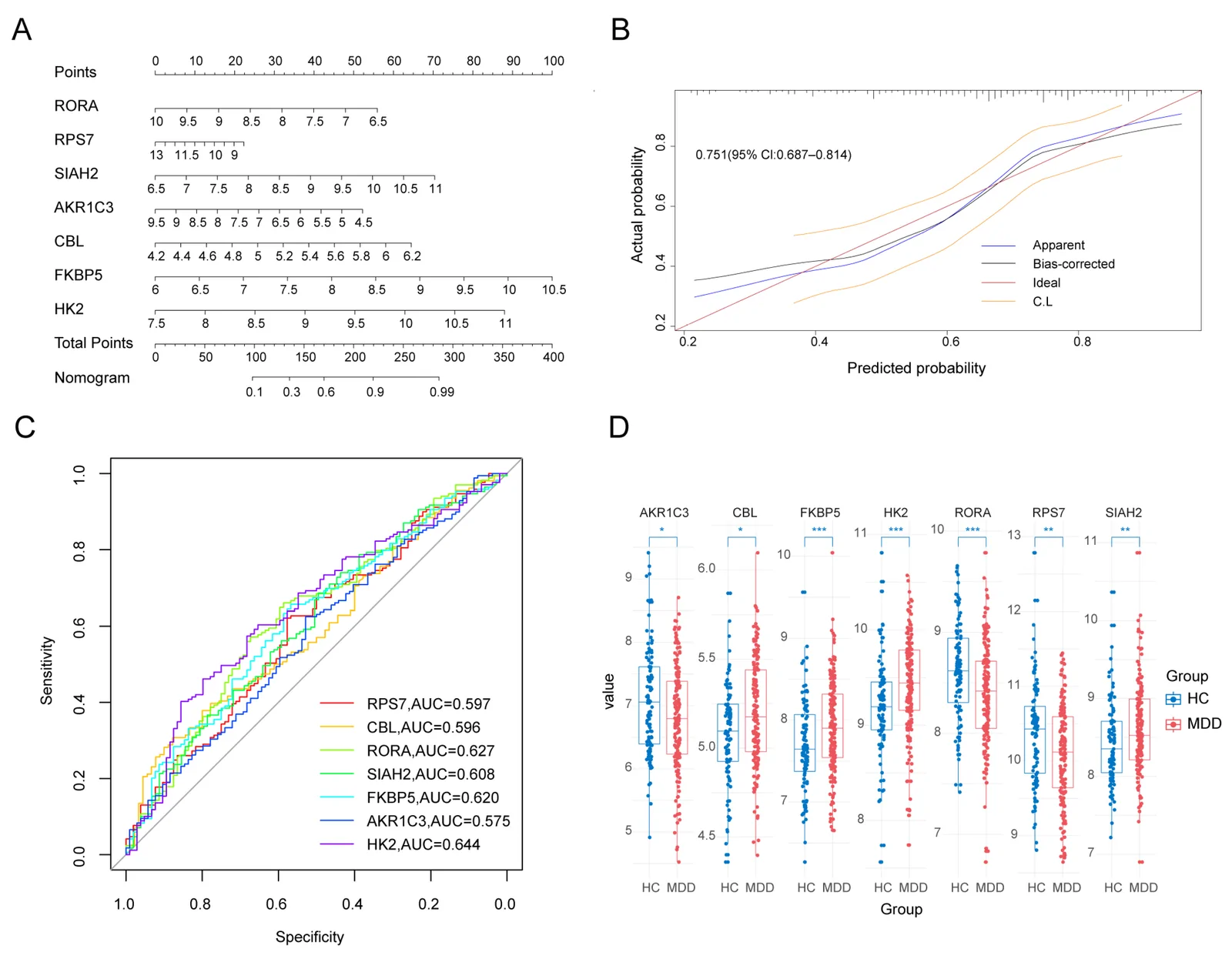

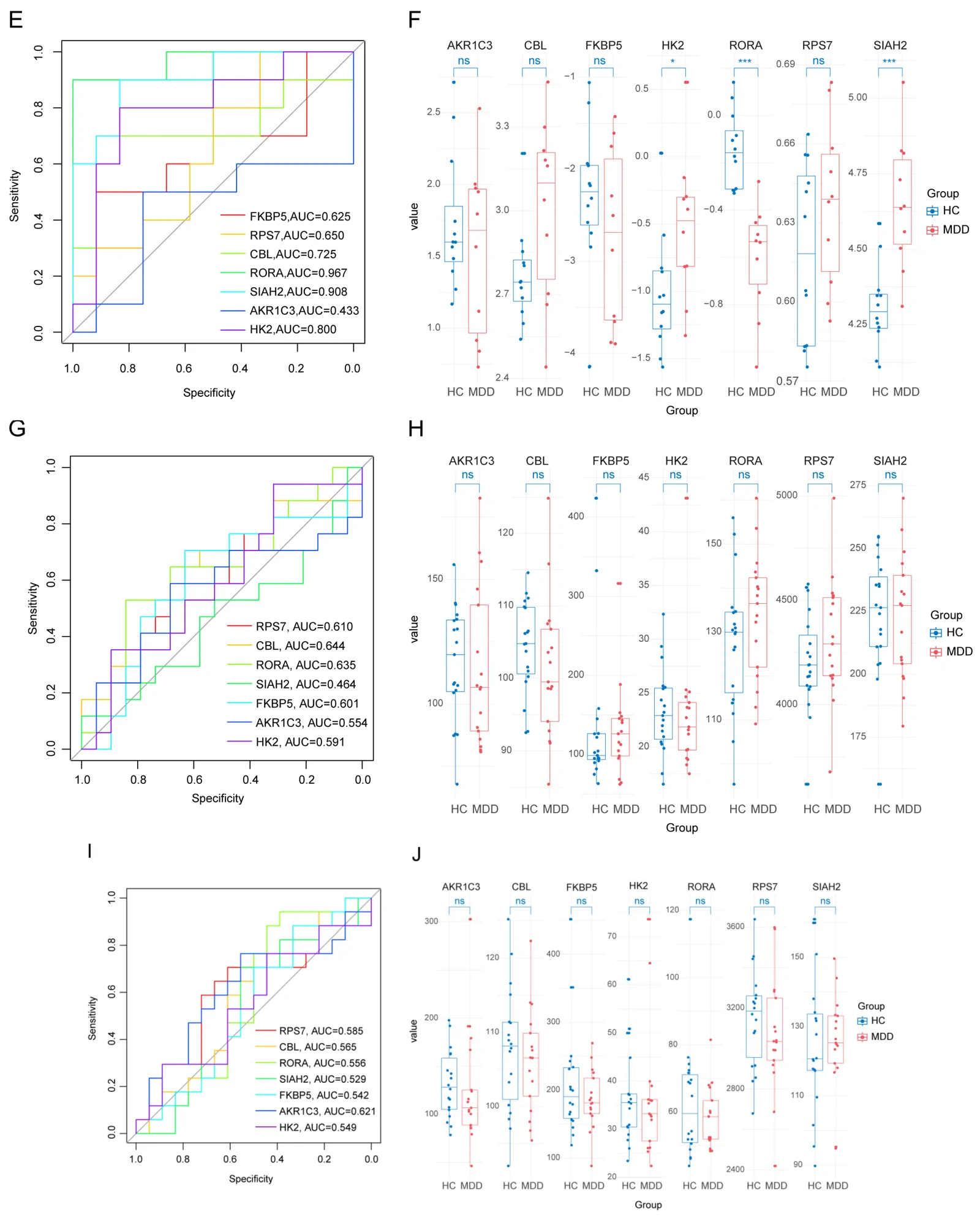
Construction and validation of predictive models for hub MD-AST DEGs. (**A**) The nomogram of the predictive model. (**B**) The calibration curves. (**C**,**D**) ROC curve and box plot of Hub MD-AST DEGs in peripheral blood from the training set. (**E**,**F**) ROC curve and box plot of Hub MD-AST DEGs in peripheral blood from the GSE76826 validation set. (**G**,**H**) ROC curve and box plot of Hub MD-AST DEGs in the PFC from the GSE53987 validation set. (**I**,**J**) ROC curve and box plot of Hub MD-AST DEGs in the HIP from the GSE53987 validation set. * *p* < 0.05, ** *p* < 0.01, and *** *p* < 0.001. ns, not significant. AUC, area under the curve. ROC, receiver operating characteristic curve.

Logistic regression analysis was performed on the hub MD-AST DEGs to construct a diagnostic model. We used a nomogram to predict the occurrence of MDD (Figure 5A) and calibration curves to verify the diagnostic efficacy of the nomogram (Figure 5B). The calibration curve analysis showed good overall calibration performance of the model, and the bias-corrected curve was close to the ideal diagonal, with only a slight probability interval deviation. The C-index was 0.751 (95% CI: 0.687–0.814), with moderately high discrimination ability, and the generalization performance was reliable after bias correction, with only a slight increase in the uncertainty of estimation in the high-probability interval. Therefore, ROC curves were plotted to validate the diagnostic performance of individual hub MD-AST DEGs and the predictive model. In the training set, HK2 exhibited the best performance, followed by RORA, SIAH2, and FKBP5; RPS7, CBL, and AKR1C3 performed moderately (Figure 5C). In the external validation set of GSE76826 (peripheral blood validation), RORA performed excellently and had near-perfect discrimination ability; SIAH2 and HK2 performed well, with strong discrimination ability; CBL, RPS7, and FKBP5 performed moderately high; and AKR1C3 performed worse than random guessing, with no practical value (Figure 5E). In the external validation set of GSE53987 (PFC validation), CBL performed the best, with moderate discrimination ability; RORA, RPS7, and FKBP5 performed moderately well; HK2’s and AKR1C3’s performances were moderately low; and SIAH2 performed worse than random guessing, lacking practical value (Figure 5G). In the external validation set of GSE53987 (HIP validation), AKR1C3 performed the best, with moderate discrimination ability, whereas all other genes exhibited moderately above-average discriminative ability (Figure 5I).

In addition, the expression levels of seven hub MD-AST DEGs were validated in the training set and the external validation sets. In the training set, FKBP5, HK2, and RORA showed the most significant differences, making them potential core biomarkers. AKR1C3 and RPS7 expressed more genes in the HC group. The expression levels of the remaining five genes were significantly higher in MDD patients than in healthy controls (*p* < 0.05), suggesting that the abnormal overexpression of these genes may be associated with Major Depressive Disorder (Figure 5D). In the external validation set GSE76826, the expression of HK2 and SIAH2 in the MDD group was significantly higher than in the HC group; the expression of RORA in the MDD group was significantly lower than in the HC group; and the expression of AKR1C3, CBL, FKBP5, and RPS7 was not statistically significant (Figure 5F). There was no statistically significant difference in the expression levels of the seven genes between HC and MDD patients in the external validation set GSE53987, indicating that they cannot independently distinguish between HC and MDD populations in the currently analyzed PFC and HIP samples, possibly requiring analysis in combination with other genes or clinical indicators (Figure 5H,J).

### 3.5. Construction of Regulatory Networks

**Figure 6:**
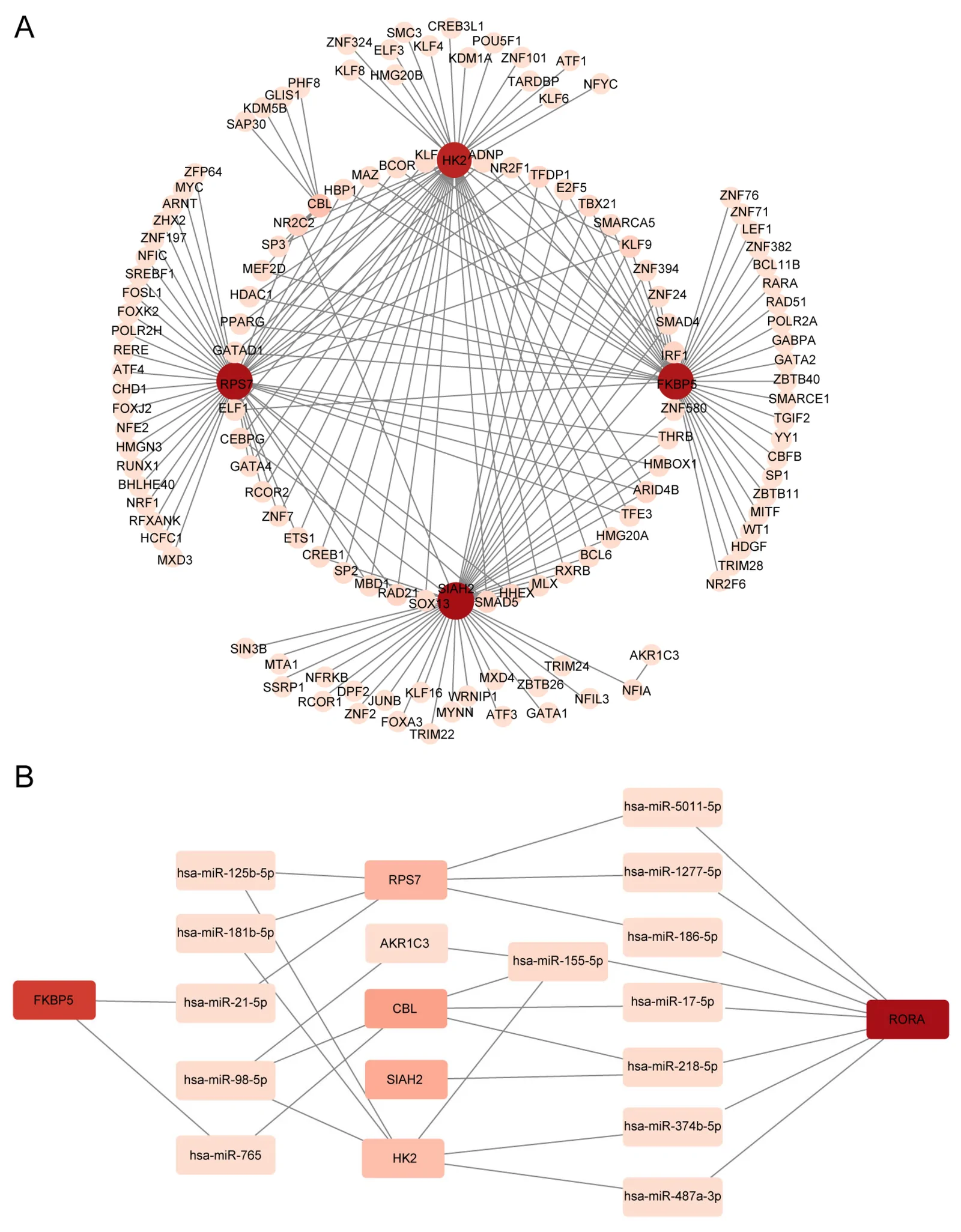
Construction of regulatory networks. (**A**) The interactions of genes and miRNA. (**B**) The interactions of TFs and genes. TF, Transcription Factor.

The TF–gene interaction network centers on HK2, RPS7, FKBP5, and SIAH2 as key hub nodes, forming dense interaction modules via numerous transcription factors, such as members of the KLF, GATA, and SMAD families (Figure 6A). The core genes exhibit a many-to-many regulatory pattern with transcription factors, suggesting potential synergistic regulatory roles in pathological pathways. The highly interconnected topology of the network also reflects the pivotal role of these core genes at the transcriptional regulatory level.

The gene–miRNA interaction network comprises 7 hub MD-AST DEGs and 13 miRNAs, revealing regulatory interactions between core target genes and their targeting miRNAs (Figure 6B). Among them, RORA serves as a central hub gene in the network, with regulatory associations with multiple miRNAs, such as hsa-miR-5011-5p and hsa-miR-1277-5p. FKBP5 is also a key node targeted by hsa-miR-21-5p and hsa-miR-1765. The network exhibits a complex many-to-many regulatory pattern, where core genes form dense regulatory modules through miRNAs, further suggesting their potential synergistic roles in pathological pathways.

### 3.6. Effects of LPS on Mouse Body Weight and Basal Motor Ability

**Figure 7:**
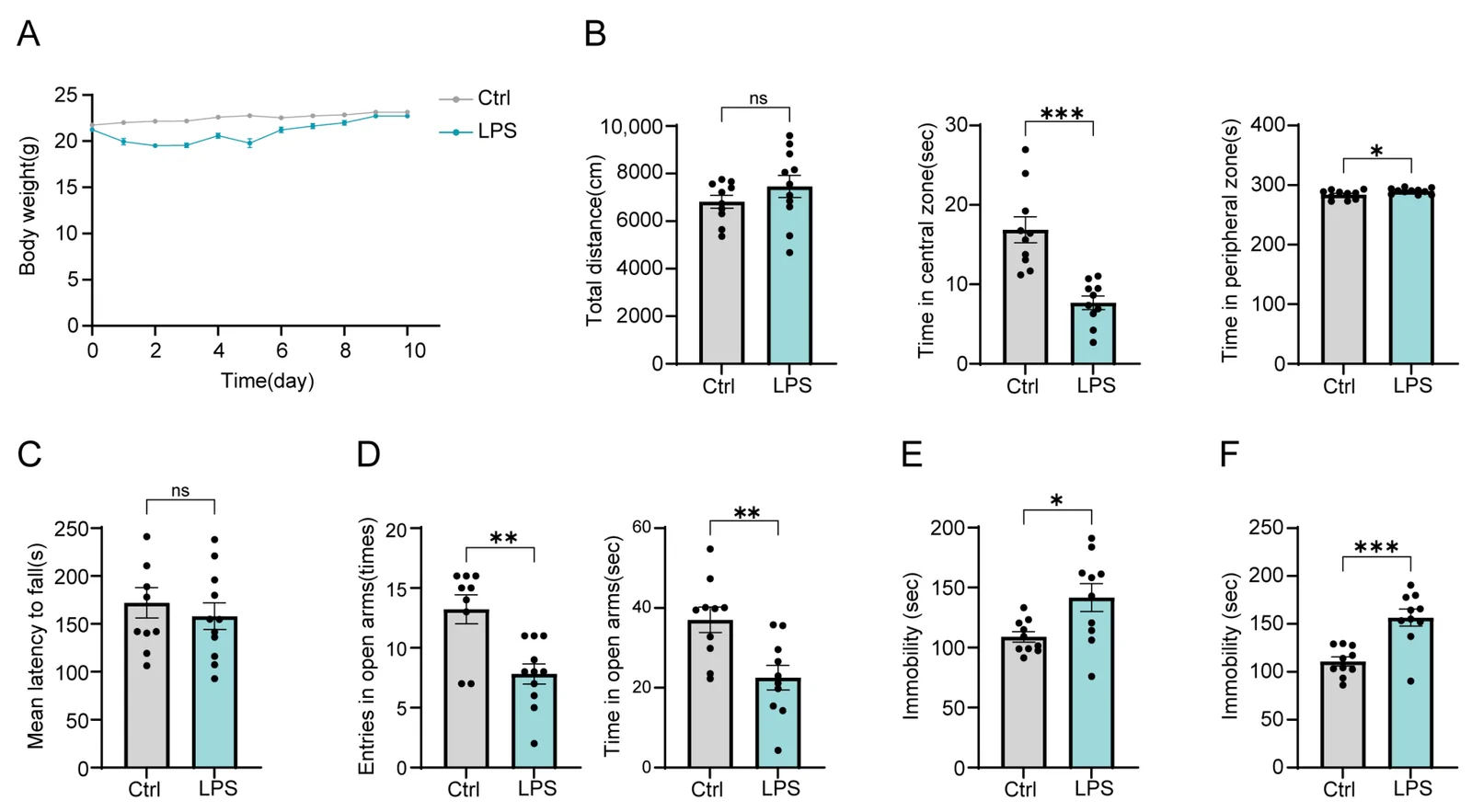
C57 mice exhibited anxiety- and depression-like behaviors following LPS injection. (**A**) Body weight. (**B**) Total distance traveled in the open field test for 30 min, and time spent in the center and peripheral zones of the open field for 5 min. (**C**) Time taken to fall in the rotarod test. (**D**) Number of entries and time spent in open arms of the elevated plus maze. (**E**) Immobility time in the tail suspension test. (**F**) Immobility time in the forced swim test. * *p* < 0.05, ** *p* < 0.01, and *** *p* < 0.001. ns, not significant.

Firstly, body weight and locomotor activity were assessed in LPS-treated mice using the OFT and the rotarod test. Similar to the control group (Ctrl), the body weight of mice in the 0.5 mg/kg LPS treatment group increased steadily over time, with no significant difference between the two groups (Figure 7A). Likewise, there were no significant differences in total distance in the OFT after 30 min, nor in the time taken to fall in the rotarod test, indicating that 0.5 mg/kg of LPS did not significantly affect the basal physiological state, spontaneous activity, or motor coordination of mice (Figure 7B,C).

### 3.7. LPS Induces Significant Anxiety-like Behavior in Mice

Anxiety-like behavior was assessed using the OFT and EPM experiments. Compared with the Ctrl group, time spent in the central area within 5 min was significantly reduced (*p* < 0.001), while time spent in the peripheral area was significantly increased in LPS mice during OFT (Figure 7B). In the EPM test, LPS mice exhibited a markedly lower number of entries into the open arms and reduced time spent in the open arms. These results indicated that 0.5 mg/kg LPS administration induced significant anxiety-like behavior (Figure 7D).

### 3.8. LPS Induces Significant Depressive-like Behavior in Mice

Depressive-like behavior was assessed using the TST and FST. As shown in Figure 7E, LPS mice exhibited a significantly increased immobility time in the TST in comparison with the control group. Consistently, a highly significant increase in immobility time was observed in LPS mice in the FST, compared with the Ctrl group (Figure 7F). These findings demonstrated that 0.5 mg/kg LPS administration led to significant depressive-like behavior.

### 3.9. Mrna Expression of Hub Genes in Lps Model Mice

**Figure 8:**
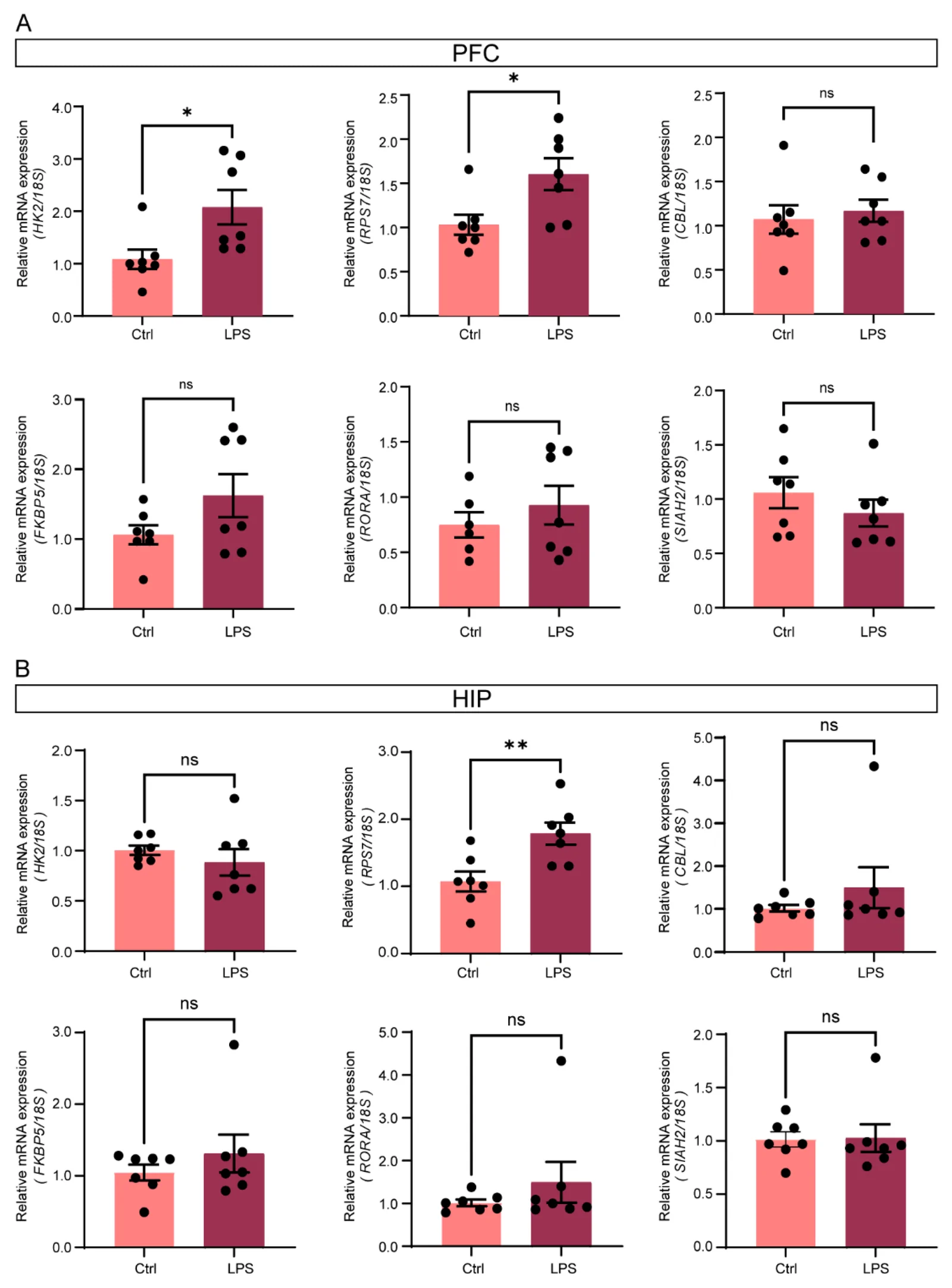
qPCR validation of 6 depression-related genes. (**A**,**B**) Quantitative polymerase chain reaction was used to detect the expression of 6 hub MD-AST DEGs (HK2, RPS7, CBL, FKBP5, RORA, and SIAH2) in the PFC and HIP of depressed mice (LPS, 0.5mg/kg, i.p., 1 week). Since mice do not have the AKRIC3 gene, qPCR detection could not be performed with this sample; therefore, only the remaining six genes were tested. * *p* < 0.05 and ** *p* < 0.01. ns, not significant. LPS, lipopolysaccharide. PFC, prefrontal cortex. HIP, hippocampus.

qPCR was performed in the LPS-induced mouse model to validate the mRNA expression levels of hub genes in vivo. Those of HK2 and RPS7 were significantly upregulated in the LPS-induced depression model compared with the control group, suggesting their potential direct involvement in LPS-mediated depressive responses. No significant differences in remaining genes’ expression were observed between the two groups, indicating that it was not markedly affected by this dosage of LPS. These genes may participate in inflammation-induced depression through alternative mechanisms or require a more robust inflammatory stimulus for activation (Figure 8A,B).

### 3.10. Protein Expression Levels of HK2 and RPS7 in LPS Model Mice

**Figure 9:**
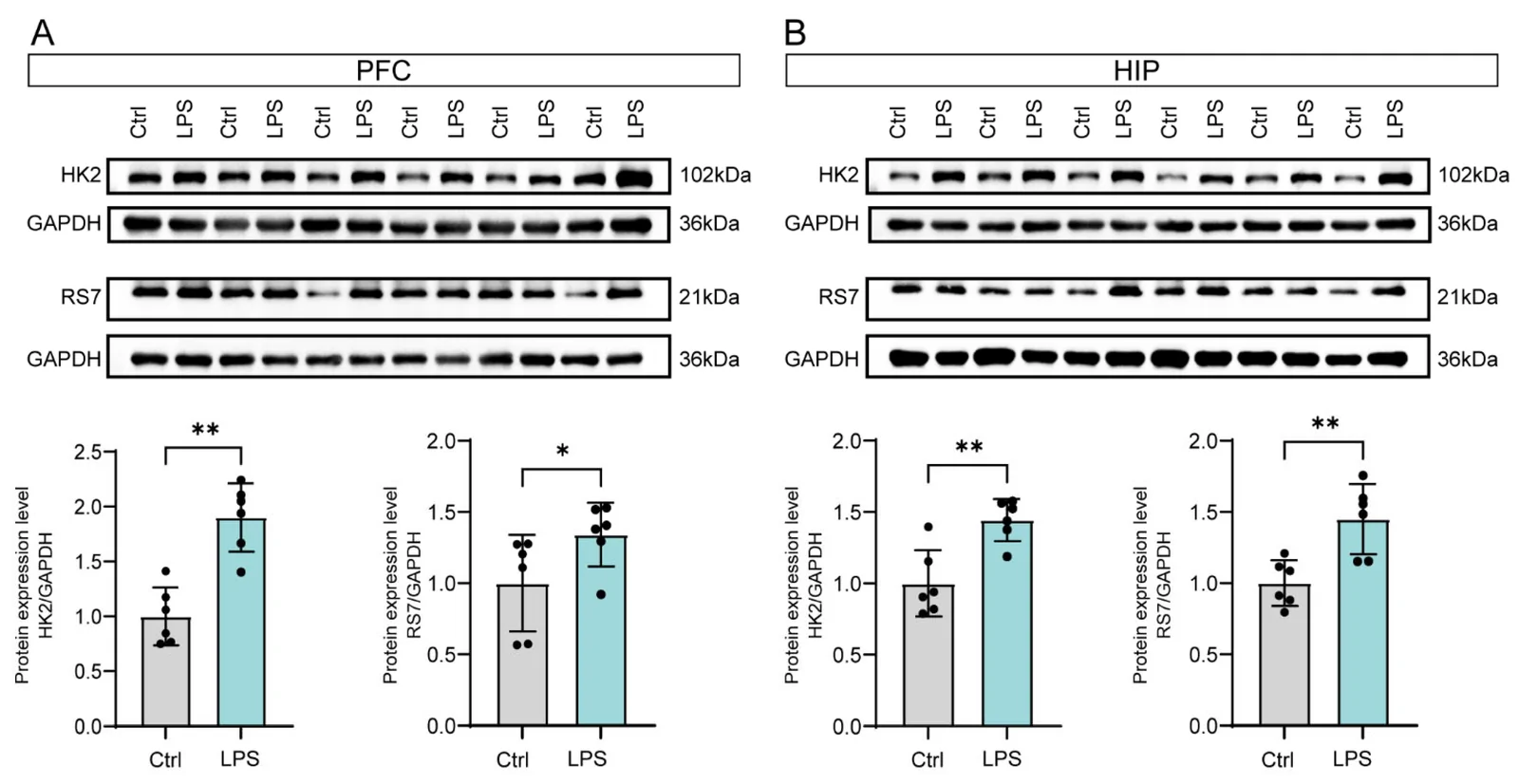
Up-regulation of HK2 and RS7 protein expression in the PFC and HIP of LPS-induced mice. Full-length blots are presented in Supplementary Figure S2. (**A**,**B**) Protein expression levels of HK2 and RS7 in the PFC and HIP (the protein corresponding to RPS7 is designated as RS7 in commercial antibodies; therefore, RS7 is uniformly used herein to refer to the Western blot results). * *p* < 0.05; ** *p* < 0.01. ns, not significant.

We sought to further validate alterations in the protein levels of HK2 and RPS7 in the PFC and HIP of LPS-induced mice; to this end, Western blot analysis was conducted. Compared with the control group, these expression levels were significantly upregulated in both the PFC and HIP of mice in the LPS-induced model group (Figure 9). The antibody for RPS7 is designated as RS7 in commercial reagents; therefore, RS7 was uniformly adopted for description in the Western blot analysis herein. The qPCR and Western blot results collectively demonstrated the ability of the LPS-induced depression model to extensively express key brain proteins associated with metabolism and stress responses (HK2 and RPS7). This upregulatory effect was present in both the PFC and HIP, suggesting that glycolytic metabolism and ribosomal function may be involved in LPS-induced neuropathological alterations.

## 4. Discussion

In this study, a strategy integrating bioinformatics analysis with animal experiments was employed to systematically screen and validate MD-AST DEGs, providing a relatively comprehensive chain of evidence for investigating the molecular mechanisms underlying depression, spanning from public database mining and machine learning modeling to in vivo experimental validation [41]. Following batch correction and principal component analysis of depression datasets from the GEO database, healthy control and depression samples could be distinctly segregated, suggesting the existence of stable and discriminative molecular expression characteristics in depression [42]. The intersection of differentially expressed genes with mitochondrial dynamics-related genes and astrocyte-associated genes yielded MD-AST DEGs, implying a close association between mitochondrial dysfunction and astrocyte disturbance in the pathogenesis and progression of depression [43,44,45]. Functional enrichment analysis revealed these genes’ primary enrichment in biological processes including the inflammatory response, energy metabolism, ribosomal synthesis, and transcriptional regulation [46,47], which are highly consistent with the classical pathological mechanisms of depression [45].

Among the eight machine learning algorithms we screened and evaluated, the SVM-RFE, RF-RFE, LASSO, and GLM demonstrated superior diagnostic efficacy, and HK2, RPS7, CBL, FKBP5, RORA, SIAH2, and AKR1C3 were ultimately identified as core hub genes. MDD is a highly polygenic disorder: the largest trans-ancestry genome-wide association study to date has identified 697 risk loci and implicated prefrontal cortical biology [48]. Against this genetic background, the seven hub genes identified in the present study may represent transcriptomic convergence points of polygenic risk, and blood-based multi-gene signatures offer a complementary, functionally proximal layer of biomarkers to DNA-level risk loci. The nomogram showed favorable discrimination in the training set and partial generalizability in external cohorts; performance was largely maintained in GSE76826 but limited in GSE53987. This suggests it as a preliminary model requiring validation in larger, prospective cohorts. The level of supporting evidence differs regarding the hub genes’ biological roles. FKBP5 has robust clinical and genetic evidence linking it to HPA-axis regulation, antidepressant response, and depression risk [49,50,51]. RPS7 and ribosomal dysregulation are supported by convergent data from human post-mortems and chronic stress in animals [52].HK2-mediated glycolytic reprogramming under neuroinflammatory conditions is supported by recent metabolic studies on neuroinflammation and depression, although direct evidence in MDD remains limited [53]. The mechanistic links of RORA, SIAH2, CBL, and AKR1C3 to depression are currently suggestive based on their annotated functions in circadian transcription, ubiquitination, signal transduction, and steroid metabolism, respectively, and remain to be experimentally established. The transcription factor and miRNA network analyses provide additional regulatory context for these genes, but these predictions are computational and require experimental validation [54].

To validate the reliability of the bioinformatics analysis results, we employed LPS to construct a mouse model of depression [55]. In behavioral assessments, mice in the LPS group exhibited significantly less time dwelling in the central zone of the open field test, fewer entries and less dwelling time in the open arms of the elevated plus maze, and significantly prolonged immobility in both the tail suspension test and forced swim test, indicating that LPS successfully induced depressive behaviors in mice. Concurrently, the rotarod test showed no significant difference between groups, excluding the confounding effect of motor performance on behavioral outcomes and ensuring the reliability of the experimental conclusions [52]. qPCR validation demonstrated significant upregulation of HK2 and RPS7 in the PFC and HIP, two critical brain regions for emotional regulation. This was consistent with the bioinformatics analysis results and further confirmed these as core genes with stably aberrant expression in depression [42,52,53]. The remaining hub genes exhibited trend-like alterations across different brain regions, suggesting a certain degree of brain region specificity in their expression. As no homologous gene for AKR1C3 exists in mice, it was excluded from detection, which is consistent with the experimental design requirements [56].

This study also observed that RPS7 exhibited down-regulated expression in bioinformatics analysis, whereas a trend toward upregulation was detected in the qPCR and Western blot results from the LPS mouse model, indicating an inconsistent expression pattern between the two approaches. The underlying reasons for this discrepancy are analyzed as follows. First, the bioinformatics data were derived from clinical samples of human patients with depression, reflecting gene expression characteristics under chronic depressive conditions, whereas this study employed an LPS-induced acute inflammatory depression model; temporal gene expression and regulatory directions differ substantially between acute stress and chronic pathological states. Second, the LPS model can only mimic inflammation-induced depression-like behavior, whereas clinical depression is highly heterogeneous in etiology, involving genetic, psychological stress, and neuroinflammatory factors; the LPS model therefore fails to fully recapitulate the pathological complexity of clinical depression. Third, interspecies differences in gene regulatory patterns exist between humans and mice; as a ribosomal protein, RPS7 demonstrates specific expression patterns across different species, brain regions, and cell types. These findings suggest that RPS7 possesses a complex regulatory mode in the pathogenesis and progression of depression, and its expression alterations are model-dependent, brain region-dependent, and species-dependent.

Notably, a recent study demonstrated that astrocyte-specific pleiotrophin deficiency in the prefrontal cortex is sufficient to induce depressive-like behaviors in male mice, providing a causal proof-of-concept that astrocyte-associated molecular changes in this brain region can drive depression-related phenotypes [34]. This finding supports the plausibility of our hypothesis that astrocyte-associated hub genes, including HK2 and RORA, may contribute to depression through similar region- and cell type-relevant mechanisms. Building on these considerations, the present findings suggest associations between the LPS-induced model and glycolytic metabolic alterations through HK2 upregulation in the PFC and HIP, as well as with ribosomal alterations via elevated RPS7 expression. Whether these molecular changes drive neuronal damage, the amplification of inflammatory circuits, and mood dysregulation remains to be established. Nevertheless, this study integrates mitochondrial dysfunction, astrocyte-associated molecular alterations, and energy metabolic disturbances into a research framework spanning from database analysis to in vivo experimental validation, providing candidate biomarkers and testable therapeutic hypotheses for further mechanistic investigation.

This study has several limitations. First, the bioinformatics analysis was based on microarray data from public databases, and the heterogeneity in sample sources, sequencing platforms, and population characteristics may have affected the generalizability of the results, along with the lack of inclusion of patients with depression from different ethnicities, age groups, and comorbidities. Second, in vivo validation was performed solely using the LPS-induced depression model, without incorporating classical depression models such as chronic unpredictable mild stress (CUMS) and learned helplessness (LH), thereby precluding a comprehensive reflection of the complex pathological processes underlying clinical depression. Third, this study only validated the expression changes in core genes (including HK2 and RPS7) at the mRNA and protein levels, lacking an in-depth exploration of their specific molecular mechanisms, upstream and downstream signaling pathways, or interaction networks. Fourth, the diagnostic prediction model for depression was only preliminarily constructed in this study, without validation in large-scale clinical cohorts; its diagnostic efficacy and clinical application value remain to be further confirmed. Fifth, only the PFC and HIP were examined, without systematic analysis of whole-brain regions; we may have overlooked important alterations in other critical brain areas. Sixth, the astrocyte association of the hub genes was inferred from database annotation (GeneCards) and validated only in whole-tissue homogenates, so astrocyte-specific expression or function cannot be concluded from the present data; cell-sorted, RiboTag, or single-cell analyses will be required for definitive validation. Additionally, gene intervention experiments were not conducted in this study, precluding the establishment of causal roles for HK2, RPS7, and other genes in depression. However, HK2-knockout experiments are currently underway. In the future, the conclusions of this study can be further refined and validated through expanded clinical samples, the construction of multiple animal models, in-depth mechanistic investigations, and intervention experiments.

## 5. Conclusions

In this study, seven core hub genes (HK2, RPS7, CBL, FKBP5, RORA, SIAH2, and AKR1C3) associated with depression were identified through bioinformatics analysis. These genes are primarily involved in biological processes such as energy metabolism, ribosomal synthesis, the inflammatory response, and transcriptional regulation. The diagnostic model constructed based on these genes exhibited favorable predictive performance and may serve as a potential auxiliary diagnostic indicator for depression. In the LPS-induced depression–anxiety mouse model, HK2 and RPS7 were significantly upregulated in the PFC and HIP, consistent with the bioinformatics analysis results, thereby confirming them as stable molecular markers for depression. The findings of this study demonstrate that abnormalities in mitochondrial dynamics, astrocyte dysfunction, enhanced glycolytic metabolism, and aberrant ribosomal synthesis collectively participate in the pathophysiological processes of depression, providing a reliable experimental foundation and novel research directions for mechanistic investigations, molecular diagnosis, and target development in depression.

## Figures and Tables

**Table 1:** RNA primer sequences.

Primer Name	Forward	Reverse
RORA	GTGGAGACAAATCGTCAGGAAT	GACATCCGACCAAACTTGACA
RPS7	AGCGCCAAGATCGTGAAGC	CACCACCAACTTCGATTTCCTT
SIAH2	CCAATGCCGCCAGAAGTTAAG	CAGGGAAACAGAACTGCCGA
CBL	AGCCCGCCTTATATCTTAGACC	AAGAGGCTGATAGTCTGCTTAGT
FKBP5	GATGAGGGCACCAGTAACAATG	CAACATCCCTTTGTAGTGGACAT
HK2	ATGATCGCCTGCTTATTCACG	CGCCTAGAAATCTCCAGAAGGG
18s	AGTTCCAGCACATTTTGCGAG	TCATCCTCCGTGAGTTCTCCA

## Data Availability

The original contributions presented in this study are included in the article and Supplementary Material. Further inquiries can be directed to the corresponding authors.

## Supplementary Materials

The following supporting information can be downloaded at https://www.mdpi.com/article/10.3390/cimb48101075/s1.
