# Supplementary material for: The Construction and Validation of a Machine Learning Model for Major Depressive Disorder Based on Astrocytes and Mitochondrial Dynamics
Source: Curr Issues Mol Biol. 2026 Oct 17;48(10):1075. doi: 10.3390/cimb48101075 (PMC13703707; doi:10.3390/cimb48101075)

Supplementary Figure 2. Full-length blots.

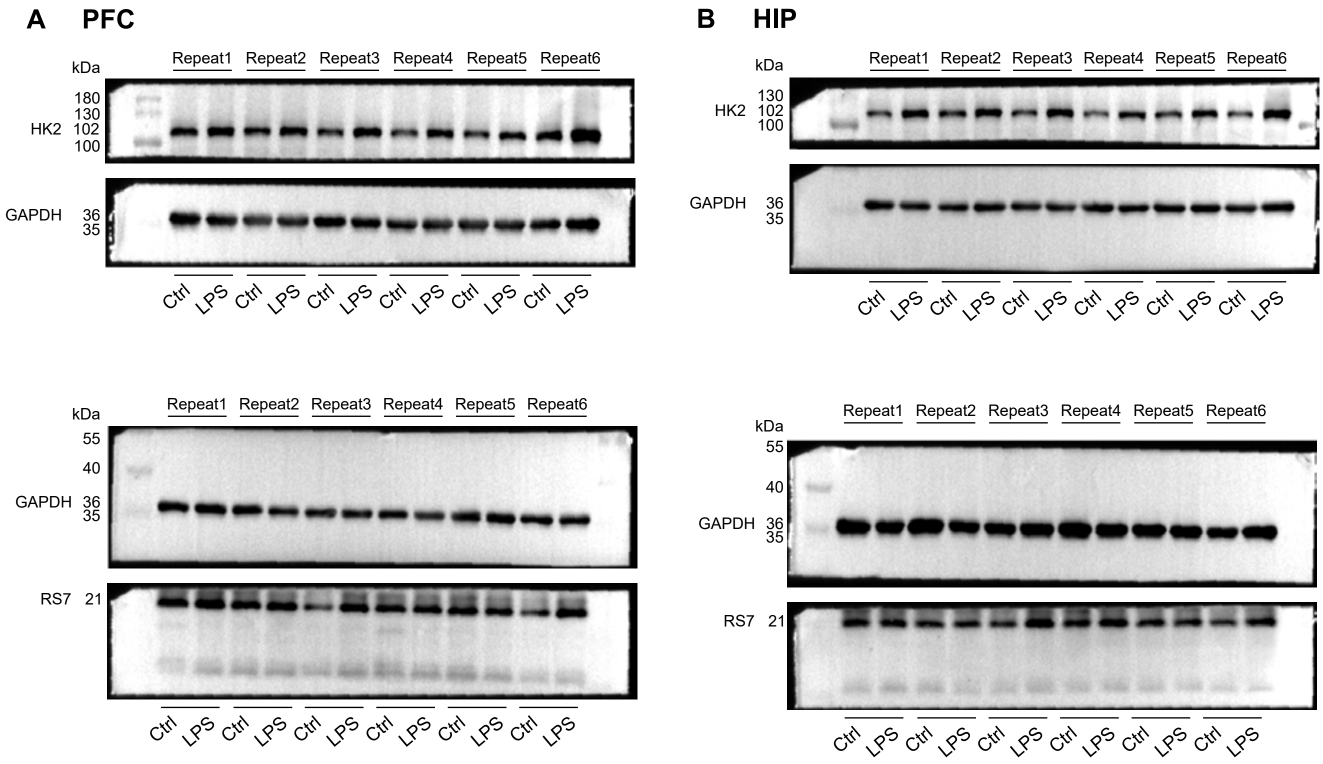

Supplement: Supplementary file 1 [file cimb-48-01075-s001.zip › Supplementary Figure S2.pdf]
